# Appearance of differentiated cells derived from polar body nuclei in the silkworm, *Bombyx mori*

**DOI:** 10.3389/fphys.2013.00235

**Published:** 2013-09-03

**Authors:** Hiroki Sakai, Takeshi Yokoyama, Hiroaki Abe, Tsuguru Fujii, Masataka G. Suzuki

**Affiliations:** ^1^Department of Integrated Biosciences, Graduate School of Frontier Sciences, The University of TokyoKashiwa, Japan; ^2^Faculty of Agriculture, Department of Biological Production, Tokyo University of Agriculture and TechnologyFuchu, Tokyo, Japan; ^3^Institute of Genetic Resources, Graduate School of Bioresource and Bioenvironmental Sciences, Kyushu UniversityHakozaki, Japan

**Keywords:** polar body nucleus, mosaic, *Bombyx mori*, short-germ band insect, serosal cell, embryogenesis

## Abstract

In *Bombyx mori*, polar body nuclei are observed until 9 h after egg lying, however, the fate of polar body nuclei remains unclear. To examine the fate of polar body nuclei, we employed a mutation of serosal cell pigmentation, pink-eyed white egg (*pe*). The heterozygous *pe*/+^*pe*^ females produced black serosal cells in white eggs, while *pe*/*pe* females did not produce black serosal cells in white eggs. These results suggest that the appearance of black serosal cells in white eggs depends on the genotype (*pe*/+^*pe*^) of the mother. Because the polar body nuclei had +^*pe*^ genes in the white eggs laid by a *pe*/+^*pe*^ female, polar body nuclei participate in development and differentiate into functional cell (serosal cells). Analyses of serosal cells pigmentation indicated that ~30% of the eggs contained polar-body-nucleus-derived cells. These results demonstrate that polar-body-nucleus-derived cells appeared at a high frequency under natural conditions. Approximately 80% of polar-body-nucleus-derived cells appeared near the anterior pole and the dorsal side, which is opposite to where embryogenesis occurs. The number of cells derived from the polar body nuclei was very low. Approximately 26% of these eggs contained only one black serosal cell. PCR-based analysis revealed that the polar-body-nucleus-derived cells disappeared in late embryonic stages (stage 25). Overall, polar-body-nuclei-derived cells were unlikely to contribute to embryos.

## Introduction

Polar bodies are small cells produced during oocyte maturation which contain one nuclei derived from the first or second meiotic division. Meiosis is generally arrested at a certain stage during oocyte maturation and the timing varies among animals (Masui, 1985). In insects, the division of an egg nucleus is arrested at metaphase during the first meiotic division (Loppin and Karr, 2005). Meiosis is resumed when the sperm enters the egg, at which time the first meiotic division is immediately completed and the first polar body nucleus formed. Then the second maturation division occurs and the second polar body nucleus expelled (Tazima, 1978). The number of polar body nucleus formed during meiosis differs between species. For example, *Pieris rapae* produces two polar body nuclei (Eastham, 1927; Tanaka, 1968). On the other hand, *Bombyx mori* (Miya, 1984), *Amata fortune* (Tanaka, 1985), *Endoclyta signifier* (Ando and Tanaka, 1980), *Drosophila melanogaster* (Campos-Ortega and Hartenstein, 1985), and Hymenoptera *Athalia rosae* (Yamamoto et al., 2008) form three polar body nuclei because the first polar body nucleus divides in synchrony with the second meiotic division.

In many animals such as mammals and echinoderms, the polar body nucleus forms from small daughter cells during meiosis and ultimately degenerates. Similarly, in *Drosophila*, polar body nuclei remain in the periplasm of oocyte during syncytial divisions and finally degenerate (Page and Orr-Weaver, 1997). However, this is not applicable to all insects. For example, in *Ageniaspis fuscicollis* (which is a variety of polyembryonic parasitoid) polar body nuclei do not degenerate and the polar-body-nuclei-derived extraembryonic membrane plays an important role in the uptake of nutrients from the host (Koscielski and Koscielski, 1985).

The fate of polar body nuclei in *B. mori* remains unclear. In this species, degeneration of the polar body nucleus does not occur during the early cleavage stage, and polar body nuclei may be observed up until 9 h after egg lying (Sato, 1926). Thus, further experiments are required in *B. mori*.

Eggs carrying polar-body-nucleus-derived cells are a type of mosaic egg. In *B*. *mori*, the appearance of mosaic egg is hardly occurred under natural conditions, however, conventional mosaic eggs (half-and-half mosaic eggs) can be induced by various artificial treatments. For example, high-temperature treatment induces (Tazima, 1939), low-temperature treatment induces (Tamazawa, 1977) and a hereditary mosaic strain, *mo*, yields mosaic eggs (Ebinuma et al., 1988).

Here, we provide the first evidence that mosaic eggs, defined as eggs with small-black spots, appear at a high frequency under natural conditions in the silkworm, *B. mori*. The presence and disappearance of the polar-body-nucleus-derived cells was also confirmed by PCR-based analyses. Our results strongly suggest that polar body nuclei can differentiate into serosal cells but seems unlikely to develop into a part of embryo.

## Materials and methods

### Silkworm colony

We used two silkworm colonies, one of which is homozygous for the *pe* gene, and the other of which is homozygous for +^*pe*^ gene. The *pe* colonies derived from National Institute of Genetics and have been maintained at the Tokyo University of Agriculture and Technology for more than 20 years. We crossed *pe*/*pe* (homozygous for the *pe* gene) individuals with +^*pe*^/+^*pe*^ (normal type for *pe*) individuals to create a colony whose genotype is +^*pe*^/*pe* (heterozygous for *pe*). In the sex-limited black egg strain 2-pB, white egg (*pe*/*pe*) is male (Z/Z) while black egg (+^*pe*^/*pe*) is female (Z/W). This strain has large chromosome composed of W chromosome, chromosome 2 bearing *p*^*B*^ gene, and chromosome 5 bearing +^*pe*^ gene (Tanaka et al., 2000). These strains have been maintained at the Tokyo University of Agriculture and Technology. Larvae were reared on fresh mulberry leaves at ~25°C.

### Microscopic observation of eggs

Eggs were observed using a stereomicroscope (SZX12, Olympus) at the diapause stage from 3 days after egg lying. Batches containing more than 20% unfertilized eggs were not observed. Microscopic images were photographed using a SCIENCE-EYE (MODEL SEYE1 30SN). In some cases, eggs were soaked in water and chorion was removed using a scalpel.

### DNA extraction and genomic PCR

DNA was extracted from the homogenized eggs using SimplePrep® reagent for DNA (Takara) according to the manufacturer's instructions. Genomic PCR was performed with EmeraldAmp® PCR Master Mix.(Takara) under the following conditions: 94°C for 2 min, 35 cycles of 98°C for 10 s, 55°C for 30 s, and 72°C for 1 min, followed by 72°C for 2 min. PCR products were analyzed by electrophoresis on 1% agarose gels and stained with ethidium bromide. To amplify the W chromosome-specific genomic DNA fragment, we used primers Rikishi-A1 (5′-GGC GAT GCT GTG TAC CCA GAA TGT-3′) and Rikishi-B2 (5′-GTT CCT CTG CGA TGG GTG GCA CAT A-3′; (Abe et al., 2005)). PCR amplification of the GAPDH gene using primers GAPDH-F(5′-CAT GAA CAG TAG TCA TCA AGC-3′) and GAPDH-R(5′-GCC GCA TTG GCC GTT TGG TGC-3′) was used as a positive control for the genomic PCR reaction.

## Results

### Analysis of serosal cell pigmentation

**Figure 1:**
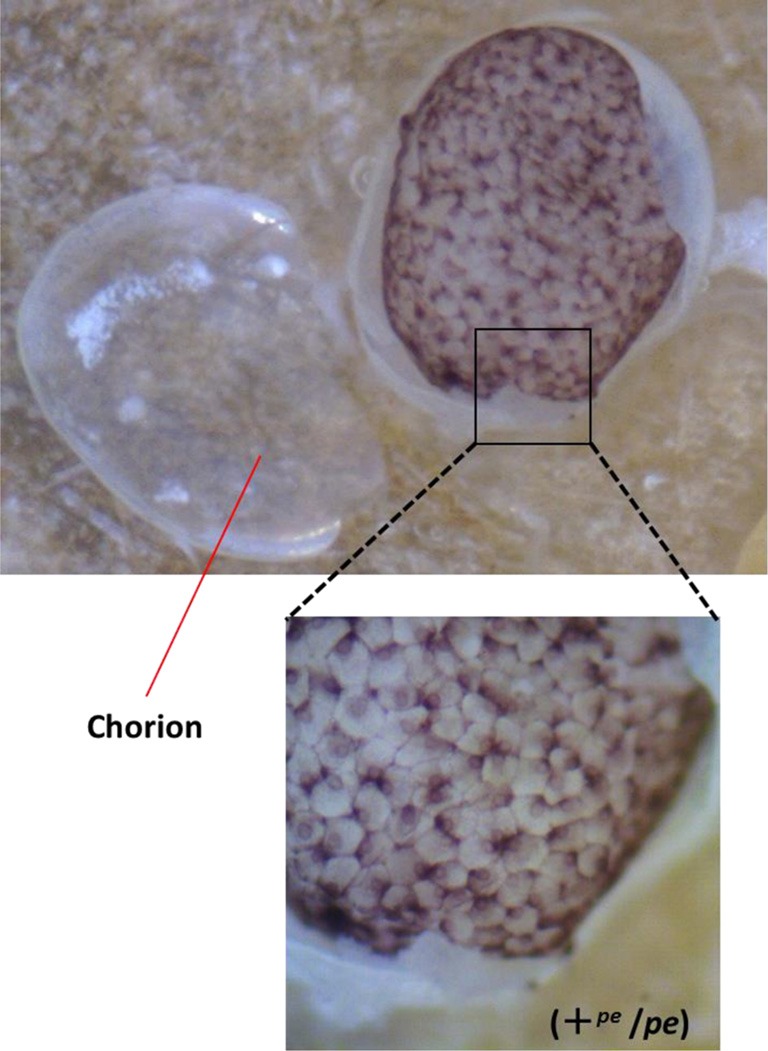
**Stereomicroscopic view of serosal cells in the black egg**. The genotype of the black egg is heterozygote for *pe* (+^*pe*^/*pe*). Chorion was removed from the egg.

**Figure 2:**
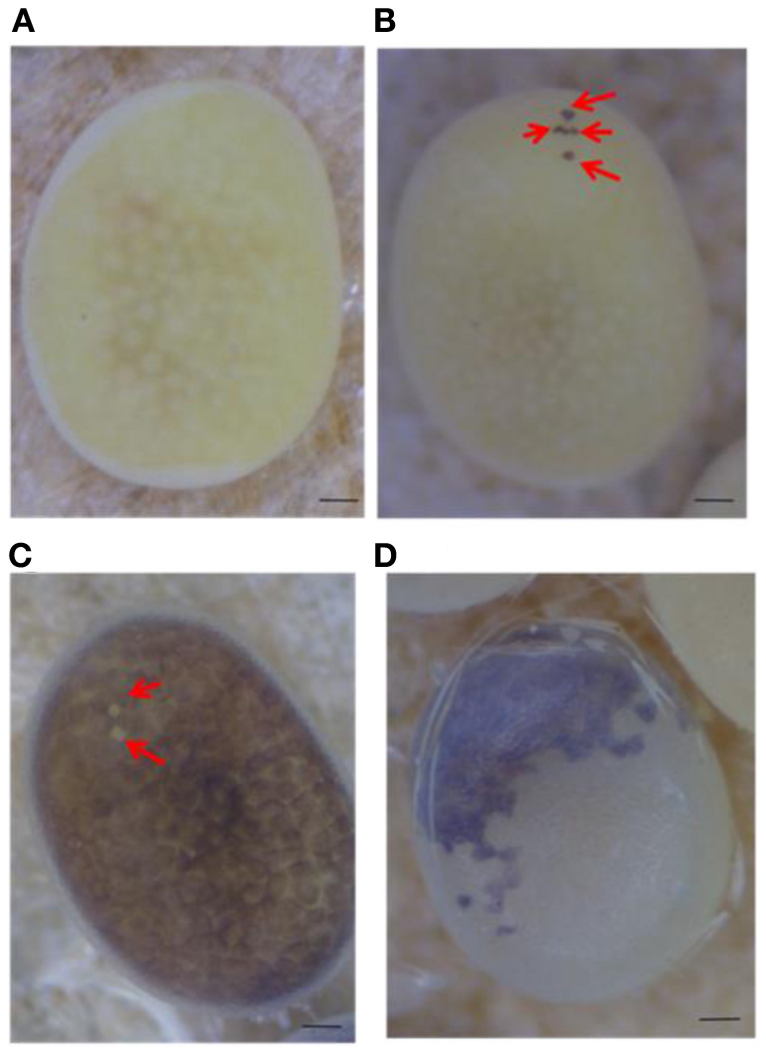
**Mosaic eggs observed in the present study. (A)** White egg laid by a *pe*/*pe* female crossed with a *pe*/+^*pe*^ male. **(B)** White egg with a small number of black serosal cells (red arrows) laid by a *pe*/+^*pe*^ female crossed with a *pe*/*pe* male. **(C)** Black egg with a small number of transparent serosal cells (red arrows) obtained from a *pe*/*pe* female crossed with a *pe*/+^*pe*^ male. **(D)** Half black and half white mosaic egg laid by a *pe*/*pe* female crossed with a *pe*/+^*pe*^ male. Scale bars represent 100 μm.

**Figure 3:**
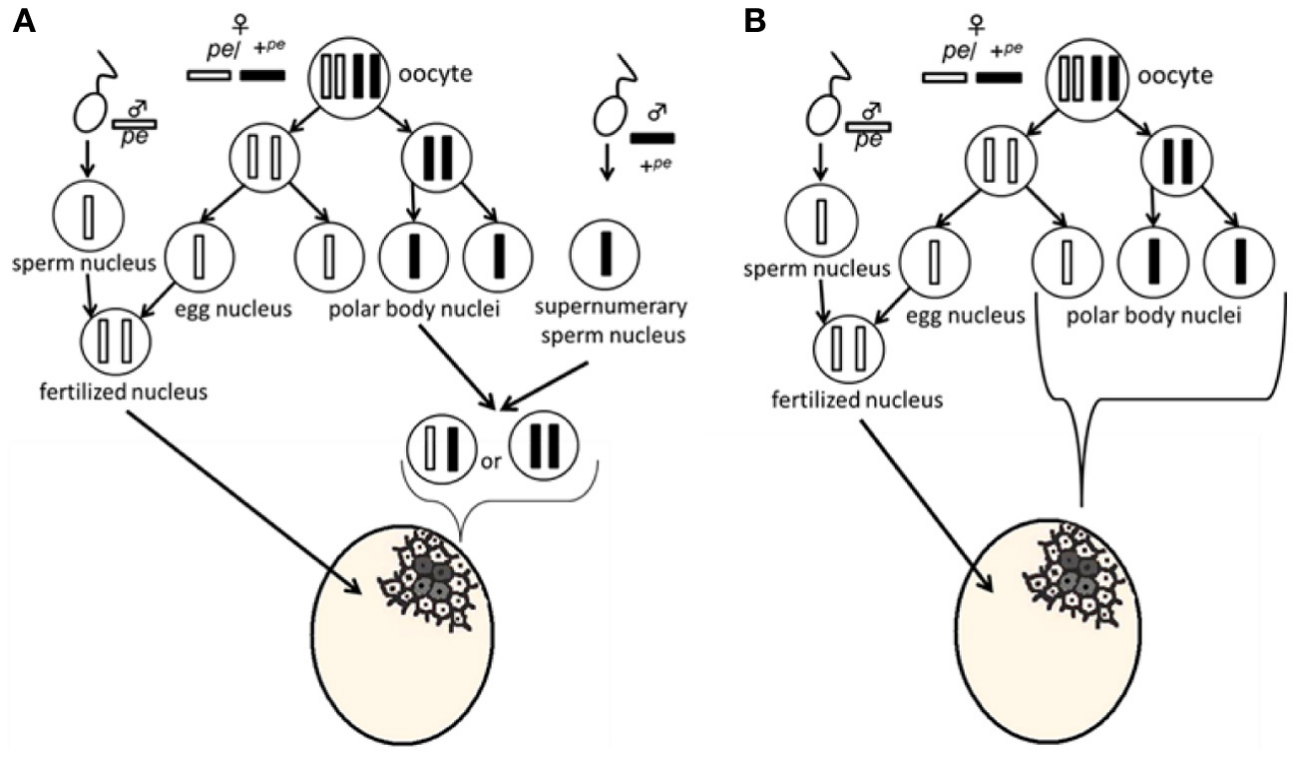
**Plausible model for the appearance of mosaic eggs**. In the white eggs laid by a *pe*/+^*pe*^ female crossed with a *pe*/*pe* male, the genotype of the fertilized nucleus is homozygous for *pe*. Meiosis proposed in this model was based on the pre-reduction model. **(A)** Most white cells come from the fusion of *pe* sperm and *pe* egg nuclei, while small black cells result from fusion of a polar body nucleus and one of the supernumerary sperm nucleus. **(B)** A single polar body nucleus or a fused polar body nucleus forms the black-spotted serosal cells.

Serosal cells constitute a single-layer membrane, lying underneath the chorion and covering the yolk and embryo. These cells are pigmented and determine egg color. Pigment synthesis occurs independently within each serosal cell. Therefore, serosal cell color can represent the genotype of each serosal cell. The extent of pigmentation and the distribution of pigment are slightly different among serosal cells, thus it is easy to distinguish identical cells from each other (Figure 1). One serosal cell color gene, *pe*, was used in our study as a recessive marker gene. The serosal cell is black when the serosal cell has the +^*pe*^ gene. We examined various crossing types as indicated in Table 1. As a result, a small number of black serosal cells were observed in white eggs (Figure 2B), and also a small number of white serosal cells were observed in black eggs (Figure 2C). In *B*. *mori*, the mosaic is defined as an individual composed of two or more cell lines that are karyotypically or genotypically distinct (Tazima, 1947). According to this definition, eggs obtained in our experiments as shown in Figure 2 were considered to be mosaic eggs. Phenotypes of mosaic eggs as shown in Figure 2D were closely resembled to those of the half-and-half mosaic eggs previously reported by Tazima (1947). The observed number of these eggs was only three among 90 batches (eggs from a single pair matings) in eggs laid by *pe*/*pe* females. Based on these results, we considered these eggs the same as the conventionally observed half-and-half mosaic eggs. In contrast, the extent of the mosaic cell population was obviously lower in the mosaic eggs shown in Figures 2B,C than the half-and-half mosaic eggs. The number of black serosal cells observed in the mosaic eggs shown in Figure 2B was ranged from 1 to 18 (Figure 5), while the number of serosal cells in the half-and-half mosaic egg shown in Figure 2D was estimated to be around 500–600. These results indicated that the mosaic eggs shown in Figures 2B,C were distinct from those previously referred mosaic eggs regarding the extent of the cells that have each genotype population. To distinguish between half-and-half mosaic eggs and the mosaic eggs shown in Figures 2B,C, we defined these as mosaic eggs with small black spots and mosaic eggs with small white spots (hereafter, black-spotted and white-spotted, respectively). The black-spotted were also appeared at the similar levels when the same experiments were performed using other serosal cell color markers, such as red-eyed red egg (*re*) or white-eyed white egg (*w-2*; data not shown). To determine the origin of the small number of black or white serosal cells observed in the small-spotted mosaic eggs, we examined various crossing types, as shown in Table 1. Because it was very difficult to precisely identify a small number of white serosal cells in a black egg, we examined black-spotted mosaic eggs. As shown in Table 1, all batches derived from a *pe*/+^*pe*^ female crossed with either a *pe*/*pe* male or a *pe*/+^*pe*^ male contained black-spotted mosaic eggs and six half black and white mosaic eggs appeared among 377 batches in eggs laid by *pe*/+^*pe*^ females. In other words, *pe*/+^*pe*^ females always produced black-spotted mosaic eggs. On the other hand, among the 112 batches, black-spotted mosaic eggs were not observed in eggs laid by *pe*/*pe* females (Table 1). Three half black and white mosaic eggs appeared among 90 batches in eggs laid by *pe*/*pe* females. These results strongly suggest that the appearance of black-spotted mosaic eggs depended on the genotype (*pe*/+^*pe*^) of the mother. Because only polar body nuclei had the +^*pe*^ gene in the white eggs laid by a *pe*/+^*pe*^ female, it is possible that the small number of black serosal cells observed in black-spotted mosaic eggs were derived from the following two origins: (1) fusion of a polar body nucleus and one of the supernumerary sperm nucleus (Figure 3A), because more than one sperm frequently enter an egg in *B. mori* (Kawaguchi, 1926), (2) a single polar body nucleus carrying +^*pe*^ or fusion of two or three polar body nuclei (Figure 3B).

**Table 1:** **The black pigmented cells in the white eggs**.

**Crossing type**		**No. of examined batches**	**No. of the batches that the normal pigmented cells were observed**
**Female genotype**	**Male genotype**		
*pe/pe*	*pe/pe*	22	0
*pe/pe*	*pe/+*^*pe*^	90	3
*pe/+*^*pe*^	*pe/+*^*pe*^	63	63
*pe/+*^*pe*^	*pe/pe*	314	314

“Batch” indicate eggs obtained from a single pair mating. Usually, about 500 eggs are contained per batch. We observed solely white eggs.

Three half black and white mosaic eggs appeared among 90 batches derived from a pe/pe female crossed with a pe/+^pe^ male and six half black and white mosaic eggs appeared among 314 batches derived from a pe/+^pe^ female crossed with a pe/pe male.

**Figure 4:**
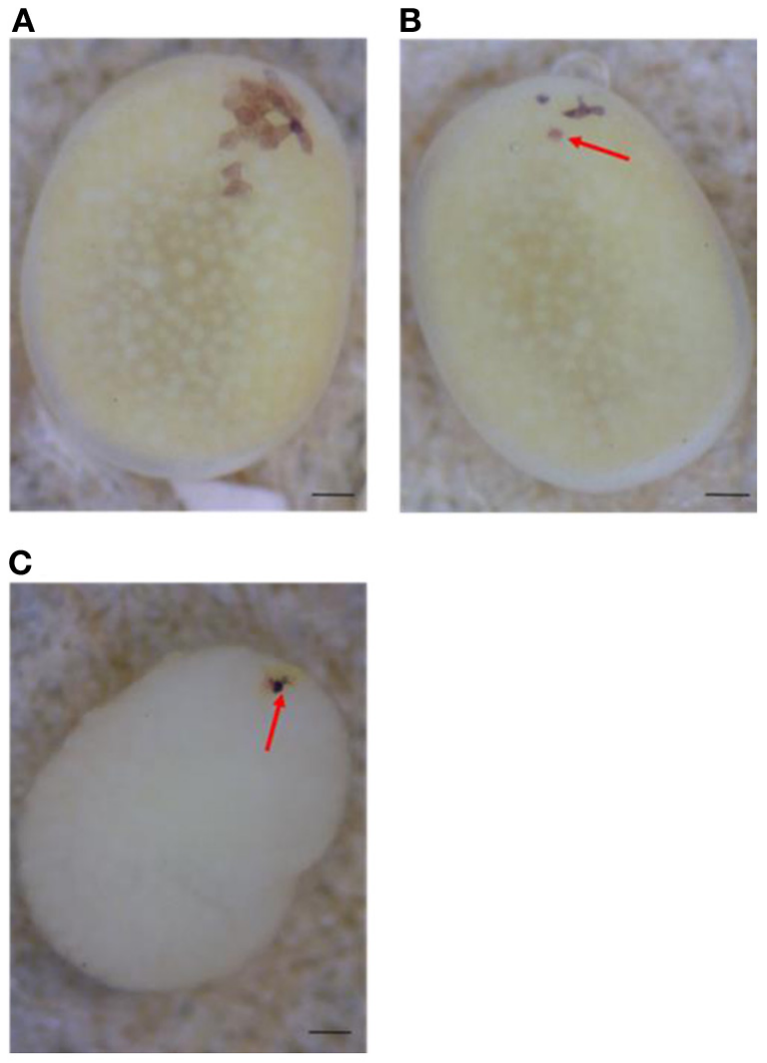
**Several forms of polar body-nucleus-derived cells laid by *pe*/+^*pe*^ females crossed with *pe*/*pe* males. (A)** White egg with a small number of fully black serosal cells. **(B)** White egg with an incompletely pigmented serosal cell (red arrow). **(C)** White egg with black granular tissues (red arrow); the chorion was removed. Scale bars represent 100 μm.

Next, to determine the origin of the small number of black serosal cells observed in black-spotted mosaic eggs, several reciprocal crossings were performed (Table 2). The half-and-half mosaic eggs did not appeared in the experiment described in Table 2. Among the 2750 white eggs obtained by crossing of *pe*/+^*pe*^ females with *pe*/*pe* males, 849 white eggs (30.8%) contained a small number of black serosal cells (Table 2). On the other hand, we found only one black-spotted mosaic egg (0.07%) among the 1383 white eggs obtained by crossing *pe*/*pe* females with *pe*/+^*pe*^ males. These results indicate that it was very rare for sperm nuclei to form the black serosal cells observed in black-spotted mosaic eggs. Therefore, the black serosal cells observed in the white eggs laid by a *pe*/+^*pe*^ female likely originated from a polar body nucleus (Figure 3B). Several forms of polar-body-nucleus-derived cells were obtained from the *pe*/+^*pe*^ female other than complete black serosal cells; for example incomplete-colored serosal cells (Figure 4B) and black granular tissues (Figure 4C). The black granular tissues did not appear to be serosal cells. However, the black granular tissues have been derived from a polar body nucleus, because they were not obtained from the *pe*/*pe* females.

**Table 2:** **The number of white eggs carrying black pigmented serosal cells**.

	**Crossing type**			**No. of White eggs**	**No. of White eggs that the normal pigmented cells were observed**
	**Female genotype**	**×**	**Male genotype**		
Batch No. 1	*pe/+*^*pe*^		*pe/pe*	268	72 (26.9%)
Batch No. 2				248	105 (42.3%)
Batch No. 3				288	113 (39.4%)
Batch No. 4				267	83 (31.1%)
Batch No. 5				250	46 (18.4%)
Batch No. 6				275	54 (19.6%)
Batch No. 7				340	97 (28.5%)
Batch No. 8				290	68 (23.4%)
Batch No. 9				211	73 (34.6%)
Batch No. 10				313	138 (44.1%)
Batch No. 11	*pe/pe*		*pe/+*^*pe*^	299	0 (0.00%)
Batch No. 12				296	0 (0.00%)
Batch No. 13				258	0 (0.00%)
Batch No. 14				267	1 (0.37%)
Batch No. 15				263	0 (0.00%)
Batch No. 16	*pe/pe*		*pe/pe*	504	0 (0.00%)

### Features of polar-body-nucleus-derived cells in white eggs

**Figure 5:**
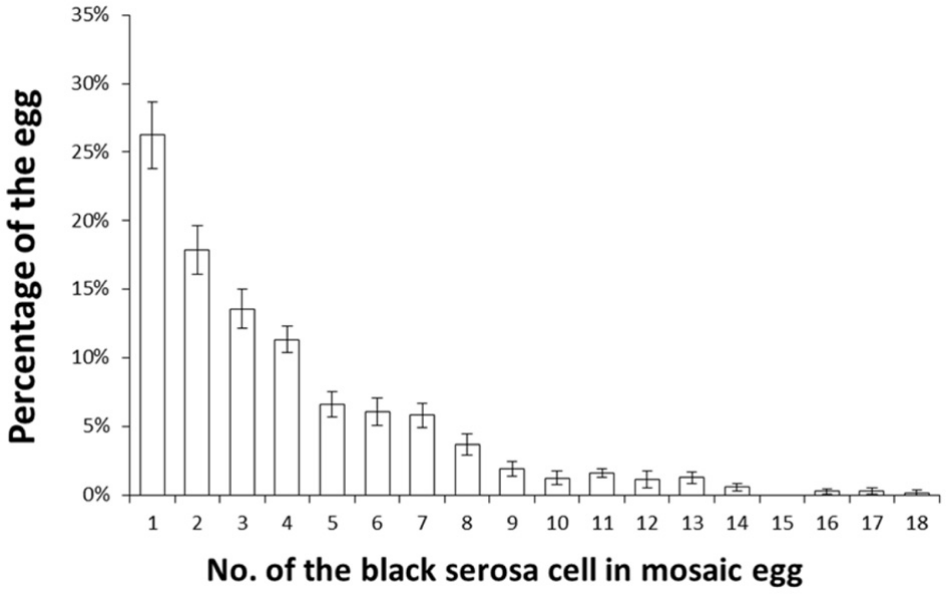
**Percentage of white eggs with the indicated number of black serosal cells**. Eleven randomly selected batches obtained from *pe*/+^*pe*^ females crossed with *pe*/*pe* males were examined. More than 100 white eggs per batch were investigated. Error bars represent standard errors. The maximum number of black serosal cells in a white egg was 18.

Next, we investigated the features of polar-body-nucleus-derived cells. We first counted the number of black serosal cells in the black-spotted mosaic eggs. Chorion was removed from an egg whose pigmented cells were difficult to count. Approximately 2.5% of white eggs formed black granular tissues, but we could not precisely estimate the number of cells within these tissues. Therefore, we decided to exclude this tissue as polar-body-nucleus-derived cells. More than 100 white eggs were randomly collected from one batch and a total of 11 batches were observed. As shown in Figure 5, the number of black serosal cells observed in the black-spotted mosaic eggs ranged from 1 to 18. Approximately 26% of mosaic eggs contained only one black serosal cell. In *B*. *mori*, three polar body nuclei formed during meiosis. Approximately 42% of the black-spotted mosaic eggs contained more than three black serosal cells. These results indicate that a polar body nucleus differentiated into serosal cells and increased its number through cell division.

Next, we investigated the positions of the black serosal cells. The egg surface was divided into four areas (anterior pole ventral side, anterior pole dorsal side, posterior pole ventral side, and posterior pole dorsal side). We referred to the swollen side as the ventral side and the shaped side as the anterior pole and observed where the black serosal cells appeared. First, we counted the number of the black serosal cell and recorded its location. We analyzed the area where the greatest number of black serosa cells were present. Of the 718 white eggs which had black serosal cells, 623 (86.8%) eggs had black serosal cells located around the anterior pole toward the dorsal side (Table 3).

**Table 3:** **The location where the black pigmented cells appear**.

**No. of white eggs which has black pigmented cells**					
	**Anterior pole**		**Posterior pole**		**Total**
	**Ventral side**	**Dorsal side**	**Ventral side**	**Dorsal side**	
Batch No. 1	2 (2.4%)	81 (97.6%)	0 (0.0%)	0 (0.0%)	83
Batch No. 2	5 (11.1%)	40 (88.9%)	0 (0.0%)	0 (0.0%)	45
Batch No. 3	2 (3.8%)	51 (96.2%)	0 (0.0%)	0 (0.0%)	53
Batch No. 4	10 (10.4%)	86 (89.6%)	0 (0.0%)	0 (0.0%)	96
Batch No. 5	17 (25.8%)	49 (74.2%)	0 (0.0%)	0 (0.0%)	66
Batch No. 6	36 (22.0%)	128 (78.0%)	0 (0.0%)	0 (0.0%)	164
Batch No. 7	19 (13.8%)	118 (85.5%)	0 (0.0%)	1 (0.7%)	138
Batch No. 8	3 (4.1%)	70 (95.9%)	0 (0.0%)	0 (0.0%)	73
Total	94 (13.1%)	623 (86.8%)	0 (0.0%)	1 (0.1%)	718

### Detection of polar-body-nucleus-derived cells by PCR

**Figure 6:**
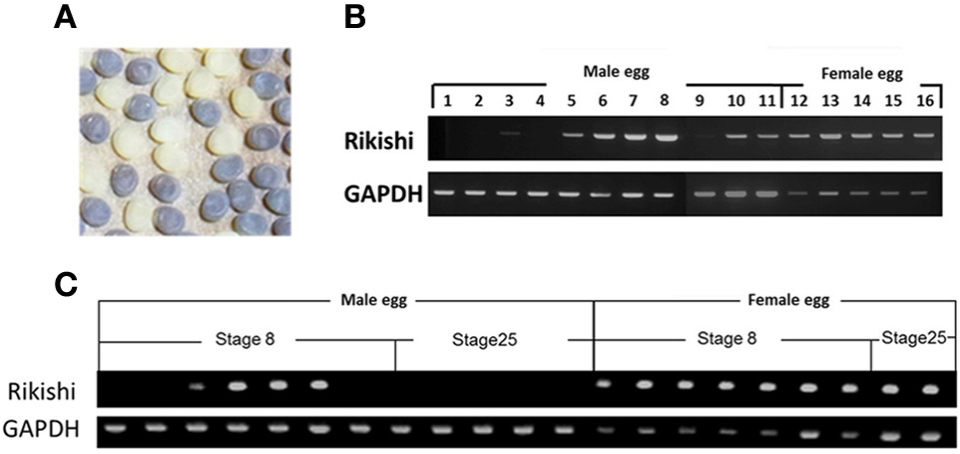
**Detection of polar body-nucleus-derived cells by PCR. (A)** In the sex-limited black egg strain 2-pB, female eggs are black (+^*pe*^/*pe*) while male eggs are white (*pe*/*pe*). **(B)** Genomic DNA extracted from each egg at stage 8 was analyzed by PCR using the W-chromosome-specific DNA marker. **(C)** The same PCR analysis as in **(B)** was performed using genomic DNA extracted from each egg at stage 25 (a taenidium in spiral bands formed in the tracheal tube at this stage) as well as stage 8.

The determination of sex in *B*. *mori* follows the ZW sex determination system; ZW for the female and ZZ for the male (Tanaka, 1916). Therefore, among three polar body nuclei, one or two polar body nuclei should have the W chromosome. If an egg contains polar-body-nucleus-derived cells, then the male eggs will have a number of W chromosomes that can be easily detected by PCR. To examine the presence and disappearance of polar-body-nucleus-derived cells, we performed PCR with genomic DNA from male egg as a template using primers amplifying the W-chromosome-specific DNA fragment. In order to discriminate male eggs from female eggs, eggs of the sex-limited strain 2-pB, in which a female was black egg and a male was white egg, were subjected to the analyses (Figure 6A). As shown in Figure 6B, the expected size of DNA was specifically amplified from all females, indicating that our PCR successfully detected the W chromosome-specific DNA. The same DNA bands were observed in six male eggs among 11 male eggs at the diaposing stage (stage8). Since the W-chromosome-specific DNA was not amplified from all male eggs, we can rule out the possibility that our PCR detects the W-chromosome-specific DNA derived from polar body nuclei which can not differentiate into cells. These results confirmed that eggs frequently contain polar-body-nucleus-derived cells under natural conditions. On the other hand, no DNA was amplified when the male eggs at stage 25 (a taenidium in the spiral band forms in the tracheal tube at this stage) were subjected to the same analysis (Figure 6C). These results demonstrated that the polar-body-nucleus-derived cells would be absent by the end of embryogenesis.

## Discussion

In *B*. *mori*, mosaic eggs and mosaic individuals can be induced either by high- (Tazima, 1939) or low-temperature treatment of fertilized eggs (Tamazawa, 1977). In addition, the hereditary mosaic strain, *mo*, yields mosaic animals at a high frequency under natural conditions (Ebinuma et al., 1988). Thus, applying special treatments and using a specific mutant strain are important to induce mosaic individuals. The appearance of mosaic eggs was thought to be rare under natural conditions. However, our results suggest that mosaic eggs appear at a high frequency under natural conditions (Table 2). Different from conventional mosaic eggs (half black and half white), the black-spotted mosaic eggs found in the present study had only a few black serosal cells (Figure 4), making them difficult to identify. This explains why these mosaic eggs have never been reported before.

Next, we found that the small number of black serosal cells observed in black-spotted mosaic eggs laid by a *pe*/+^*pe*^ female originated from a polar body nucleus. In addition, it was very rare for sperm nuclei to participate in the formation of black serosal cells observed in these eggs (Table 2). In this context, artificial induction of androgenesis is prohibitively difficult to yield fully developed embryos compared to parthenogenesis (Tamazawa, 1977; Sugai et al., 1987). In fact, the hatchability of androgenetically activated eggs was very low, less than 1% (Sugai et al., 1987). On the other hand, the hatchability of parthenogenetically activated eggs was ~70% (Sugai et al., 1983). The sperm nuclei could participate in development only when they fused with egg nuclei. These findings demonstrate that the sperm nuclei alone have little or no potential to participate in development and complete normal embryogenesis compared to the egg nucleus in natural conditions. Moreover, eggs deposited by *l-mo* (mosaic lethal) females die during embryogenesis because supernumerary sperm nuclei begin an androgenetic mode of development (Goto and Kobayashi, 1997). The result indicated it is important for embryogenesis to inactivate supernumerary sperm nuclei.

In *B. mori*, an egg nucleus just after oviposition is arrested at metaphase of the first meiotic division, which is released by egg activation and the first polar body nuclei is produced. The second meiotic division takes place about 60 min after oviposition, and maturation of the egg nucleus is completed (Tazima, 1978). The first polar body nucleus division occurs at the same time as the second maturation division, yielding three polar body nuclei (Sato, 1926). Two or three polar body nuclei are fused and subsequent divisions occur in the periplasm. Nuclei derived from polar body nuclei are observed until 9 h after oviposition (Sato, 1926). However, after the blastula stage, the fate of the polar body nucleus remains unknown. Our findings indicate that these polar body nuclei can survive and have the ability to differentiate into serosal cells. As shown in Table 3, polar-body-nucleus-derived serosal cells appeared near the anterior pole and the dorsal side. The meiotic divisions in *Bombyx* eggs mainly occur in the anterior dorsal periplasm of the egg (Miya, 1984). Therefore, the distribution of the pigmented serosal cells to the opposite area of germ band formation may simply result from the division of polar body nuclei originally located near the anterior dorsal periplasm. In *B. mori*, embryogenesis occurs in the posterior pole and the ventral side. This suggests that the polar-body-nucleus-derived serosal cells are formed opposite to where embryogenesis occurs. In addition, the number of polar-body-nucleus-derived serosal cells was low (Figure 5), and the W-chromosome-specific DNA fragment was not detected in any male eggs in later embryonic stages (Figure 6C). Overall, it seems unlikely that polar-body-nucleus-derived cells developed into part of the embryo.

The biological importance of the polar body nucleus for insects remains unclear. For *Drosophila*, which has a long germ band, three polar body nuclei develop during meiosis, and these polar body nuclei stay in the surface layer of the egg during syncytial blastoderm stage (Campos-Ortega and Hartenstein, 1985). A previous study reported that BubR1, which is located in the centromere and controls cell cycle checkpoints, is seen in the chromosome of the polar body nuclei. Interestingly, the polar body nucleus in the BubR1 mutant can synthesize DNA (Pérez-Mongiovi et al., 2005). This supports the concept that the polar body nucleus retains mitotic activity, and its activity is inactivated by specific inhibitory mechanisms.

In insects with long-germ embryos, almost all parts of the blastoderm develop into the embryo. Therefore, some mechanisms may be required to inactivate polar body nuclei and prevent their incorporation into embryonic cells. On the other hand, in insects that form short-germ embryos (which are relatively ancestral), a small part of the blastoderm develops into an embryo. In the present study, polar-body-nucleus-derived serosal cells were observed in nearly half of the eggs in *B. mori*, which is an example of a short-germ insect. Our findings support the possibility that inactivation and degradation of polar body nuclei are not necessarily important during the early developmental stage for normal embryogenesis in *B. mori*. In the beetle *Tribolium castaneum*, which is also a short-germ insect, the polar body nucleus is closely associated with the cortical microtubule network, which may play a role in the localization of maternal transcripts during oogenesis (Peel and Averof, 2010). Therefore, the polar body nucleus may be essential for early development in short-germ insects. Further studies are required to determine whether polar-body-nucleus-derived cells play important roles in normal embryogenesis in *B. mori*.

### Conflict of interest statement

The authors declare that the research was conducted in the absence of any commercial or financial relationships that could be construed as a potential conflict of interest.
